# FGFR4 Gene Polymorphism Reduces the Risk of Distant Metastasis in Lung Adenocarcinoma in Taiwan

**DOI:** 10.3390/ijerph17165694

**Published:** 2020-08-06

**Authors:** Ju-Pi Li, Hsien-Cheng Huang, Po-Jen Yang, Chien-Yuan Chang, Yu-Hua Chao, Thomas Chang-Yao Tsao, Hsuan Huang, Yu-Ching Hung, Ming-Ju Hsieh, Shun-Fa Yang

**Affiliations:** 1 School of Medicine, Chung Shan Medical University, Taichung 402, Taiwan; d888203@gmail.com (J.-P.L.); cshy1030@csh.org.tw (P.-J.Y.); nka6150@gmail.com (Y.-H.C.); his885889@gmail.com (T.C.-Y.T.); 2 Department of Pediatrics, Chung Shan Medical University Hospital, Taichung 402, Taiwan; 3 Department of Emergency Medicine, Kuang Tien General Hospital, Taichung 433, Taiwan; asiantcumed@gmail.com; 4 Department of Family and Community Medicine, Chung Shan Medical University Hospital, Taichung 402, Taiwan; 5 Institute of Medicine, Chung Shan Medical University, Taichung 402, Taiwan; 6 Petite Doris Clinic, Taichung 408, Taiwan; Darong14@gmail.com; 7 Division of Chest, Department of Internal Medicine, Chung Shan Medical University Hospital, Taichung 402, Taiwan; 8 School of Medical Laboratory and Biotechnology, Chung Shan Medical University, Taichung 402, Taiwan; boss871215@icloud.com (H.H.); bery830746@gmail.com (Y.-C.H.); 9 Cancer Research Center, Changhua Christian Hospital, Changhua 500, Taiwan; 10 Graduate Institute of Biomedical Sciences, China Medical University, Taichung 404, Taiwan; 11 Department of Medical Research, Chung Shan Medical University Hospital, Taichung 402, Taiwan

**Keywords:** lung cancer, FGFR4, polymorphism, EGFR mutation

## Abstract

Fibroblast growth factor receptor 4 (*FGFR4)* is involved in multiple physiological and pathological processes. Several genetic variants of *FGFR4* have been shown to be associated with tumor progression in many cancers. However, its association, such as genetic variants and expression levels, with lung cancer is controversial. The present study examined the relationship between four single-nucleotide polymorphisms (SNPs; rs2011077 T/C, rs351855 G/A, rs7708357 G/A, and rs1966265 A/G) of *FGFR4* and the risk of lung adenocarcinoma with the epidermal growth factor receptor (*EGFR*) mutation status in a Taiwanese cohort. The results demonstrated that *FGFR4* rs2011077 (odds ratio (OR) = 0.348, 95% confidence interval (CI) = 0.136–0.891, *p* = 0.024), and rs351855 (OR = 0.296, 95% CI = 0.116–0.751, *p* = 0.008) showed an inverse association with distant metastasis in wild-type *EGFR* lung adenocarcinoma. Furthermore, a database analysis using The Cancer Genome Atlas revealed that the higher *FGFR4* expression level was correlated with poor survival rates in wild-type *EGFR* lung adenocarcinoma. In conclusion, the data suggest that *FGFR4* SNPs may help in identifying patient subgroups at low-risk for tumor metastasis, among carriers of lung adenocarcinoma bearing wild-type *EGFR*.

## 1. Introduction

Lung cancer has the highest global incidence and mortality rate of all cancers [1]. According to statistical data from Taiwan’s Ministry of Health and Welfare, the leading causes of cancer-related death in 2018 were tracheal, bronchial, and lung cancer. Lung adenocarcinoma, accounting for approximately 50% of lung cancer cases, occurs frequently in the female and nonsmoking populations [2,3]. In addition to environmental risk factors such as smoking and exposure to certain chemicals, genetic variation also increases the risk of lung cancer. Up to 30% of patients with lung adenocarcinoma in the Asian population have been shown to harbor epidermal growth factor receptor (*EGFR*) mutations [4,5]. Although *EGFR* inhibitors are highly efficacious in treating lung cancer, not all lung adenocarcinomas respond to this therapy. Therefore, the identification of other biomarkers and novel treatment strategies for lung adenocarcinoma is an unmet need.

Fibroblast growth factor receptors (*FGFR*s), which comprise five members (*FGFR1*-5), belong to the receptor tyrosine kinase family [6]. *FGFR*s participate in multiple physiological and pathological processes, such as proliferation, differentiation, metabolism, and migration [6]. Dysregulation of *FGFR* signaling cascades has been shown to be highly associated with tumor development in numerous human cancers [7]. Single-nucleotide polymorphisms (SNPs), the most common type of genetic variation, are reported to be significantly associated with cancer morbidity and mortality [8]. Numerous SNPs in *FGFR* genes, including *FGFR4*, have been identified as being significantly associated with cancer susceptibility and progression [7,9]. The human *FGFR4* gene is located on chromosomal locus 5q35 and contains 18 exons [10]. Recent studies have focused on the clinical significance of *FGFR4* genetic variations [10]. *FGFR4* SNPs have been shown to be significantly associated with poor survival rates and worse prognosis in prostate cancer [11,12], head and neck cancer [13,14], breast cancer [15,16,17,18,19], ovarian cancer [20], hepatocellular carcinoma [21], uterine cervical cancer [22], urothelial cell carcinoma [23], pituitary tumors [24,25], soft tissue sarcoma [26], and oral squamous cell carcinoma [27,28,29]. However, the effects of *FGFR4* SNPs on the risk of lung cancer seem to be controversial. The most common *FGFR4* genetic variant in lung cancer is the SNP rs351855 G/A, which is in the exon 9 coding region of the *FGFR4* gene. The *FGFR4* SNP rs351855 induces the substitution of glycine for arginine at codon 388 (G388R) in the transmembrane domain of the FGFR4 protein receptor [30,31]. The FGFR4-G388R SNP has been shown to be significantly correlated with poor outcomes, including lymph node involvement and reduced overall survival [32,33], and is associated with worse survival in node-positive patients [34]. Conversely, this SNP was also reportedly correlated with better outcomes, including longer overall survival and reduced cancer risk in patients with non-small-cell lung carcinoma [35]. In addition, no association between *FGFR4* variants and clinical outcomes has been found [36]. These conflicting results require further examination using a greater variety of research designs.

The role of *FGFR4* gene polymorphisms in the clinicopathological characteristics of lung adenocarcinoma in the Taiwanese cohort remains unclear. The prevalence of *EGFR* mutations is relatively high in the Taiwanese population with lung adenocarcinoma [2,4]. Therefore, we examined the association between the *FGFR4* SNPs and *EGFR* mutation in Taiwanese patients with lung adenocarcinoma. We explored four SNPs of *FGFR4*, namely rs2011077, rs351855, rs7708357, and rs1966265, to examine the pathophysiological effects on tumorigenesis of several cancers. Furthermore, we studied the effect of *FGFR4* SNPs on the clinicopathological characteristics of lung adenocarcinoma with and without *EGFR* mutation.

## 2. Materials and Methods

### 2.1. Participant Selection and Specimen Collection

This study included 277 patients with lung adenocarcinoma at Cheng-Ching General Hospital in Taichung, Taiwan between 2012 and 2015. Each patient provided signed informed consent before initiation of the study. From all participants, tumor tissue specimens and whole-blood specimens were collected for *EGFR* gene sequencing and *FGFR4* gene polymorphism analysis, respectively. The clinical information of the enrolled patients and the examined lifestyle variables (e.g., cigarette smoking) were obtained from medical records and questionnaires, respectively. The adenocarcinoma was staged in accordance with the TNM Classification of Malignant Tumors (where TNM refers to tumor/node/metastasis) of the American Joint Committee on Cancer, where T1–T4 describes primary tumor sizes and the invasion of adjacent tissues, N describes the regional lymph nodes involved, and M describes distant metastasis [37]. The study protocol was approved by the Institutional Review Board of Cheng-Ching General Hospital (No. HP120009; 22 September 2012).

### 2.2. Genomic DNA Extraction and EGFR Gene Sequencing

Genomic DNA from tumor tissues and whole-blood specimens of patients with lung adenocarcinoma were prepared as described previously [38]. DNA was extracted using the QIAamp Fast DNA Tissue Kit and the QIAamp DNA Blood Mini Kit (Qiagen, Valencia, CA, USA) according to the manufacturer’s instructions. DNA from the tumor tissues was used as a template. Exons 18–21 of the EGFR gene were amplified using the polymerase chain reaction (PCR) and then subjected to the DNA sequencing reaction using the ABI PRISM 3130XL System (Applied Biosystems, Foster City, CA, USA) as described previously [3].

### 2.3. Genotyping of FGFR4 Polymorphisms

The four SNPs investigated, namely rs2011077, rs351855, rs7708357, and rs1966265, in the *FGFR4* genome region have been well defined and suggested to be associated with the risk of various cancers susceptibility [22,23,29,39]. The peripheral blood samples from lung cancer patients were collected for genomic DNA extraction. The whole blood samples were placed in EDTA containing tubes and were centrifuged at 3000 rpm, for 10 min as described previously [40]. Real-time PCR genotyping with the TaqMan SNP Genotyping Assay and the ABI StepOne Real-Time PCR System were used to examine the SNPs (Applied Biosystems, Foster City, CA, USA).

### 2.4. Statistical Analysis

Differences in the distributions of clinical characteristics and genotype frequencies between patients with lung adenocarcinoma harboring wild-type *EGFR* and mutant-type *EGFR* were analyzed using the Mann–Whitney U test and Fisher’s exact test. The association between the genotype frequencies and risk of *EGFR* types was estimated using multiple logistic regression models after controlling for other variables and is presented as odds ratios (ORs) with corresponding 95% confidence intervals (CIs). A *p*-value of <0.05 was considered to be statistically significant. All analyses were generated using SAS software, version 9.1 of the SAS System (SAS Institute Inc., Cary, NC, USA).

## 3. Results

### 3.1. Characteristics of the Study Population

A total of 277 patients with lung adenocarcinoma, divided into those harboring wild-type *EGFR* (*n* = 108, 40%) and those harboring mutant-type *EGFR* (*n* = 169, 60%) were included in this study. Table 1 shows the clinical characteristics of the enrolled patients. As Table 1 shows, the two groups, the wild-type *EGFR* group and the mutant-type *EGFR* group, differed significantly with respect to gender (*p* < 0.001), cigarette smoking (*p* < 0.001), and cell differentiation (*p* = 0.002). The *EGFR* mutant-type group had a higher proportion of women (*n* = 109, 64.5%), nonsmokers (*n* = 131, 77.5%), and cases of well differentiated (*n* = 21, 12.4%) and moderately differentiated tumors (*n* = 138, 81.7%) compared with the *EGFR* wild-type group (Table 1). The other clinicopathological characteristics, such as tumor stage, tumor T status, lymph node status, and distant metastasis, were similar between groups (Table 1).

### 3.2. Genotype Distributions of FGFR4 Polymorphisms in Patients with Lung Adenocarcinoma

To explore the effects of *FGFR4* polymorphisms on lung adenocarcinoma risk, a whole-blood specimen from each of the 277 patients was collected and examined for genotype frequencies of the four *FGFR4* SNPs (rs2011077, rs351855, rs7708357, and rs1966265). The genotype distributions and associations between the four *FGFR4* polymorphisms and lung adenocarcinoma are shown in Table 2. Among the patients, the alleles with the highest distribution frequency for rs2011077, rs351855, rs7708357, and rs1966265 were heterozygous for T/C, heterozygous for G/A, homozygous for G/G, and heterozygous for A/G, respectively. To reduce the influence of possible confounding variables, we used adjusted ORs with 95% CIs—estimated using the multiple logistic regression model after normalizing for age, gender, and smoking habits—to evaluate the distribution of SNPs. As Table 2 shows, no significant differences in the genotype distributions of the four *FGFR4* SNPs between patients with wild-type *EGFR* versus those with mutant-type *EGFR* lung adenocarcinoma were observed.

### 3.3. Associations between FGFR4 SNPs rs2011077 and rs351855 and Distant Metastasis among Lung Adenocarcinoma Patients with the Wild-Type EGFR Gene

Next, we examined the genotype frequencies of each of the four *FGFR4* SNPs and the clinicopathological characteristics of the patients: Whole tumor stage, tumor T status (primary tumor size), lymph node status, existence of distant metastasis, and cell differentiation (the histopathological grading status). As Table 3 shows, we observed an inverse association between *FGFR4* rs2011077 and distant metastasis, although it was not significant (*n* = 277, *p* = 0.061). We further performed a subgroup analysis based on the EGFR status: Wild-type (*n* = 108) and mutant-type (*n* = 169). Compared with the homozygous TT genotype, the existence of at least one allele of SNP rs2011077 C genotype (TC and CC) was associated with a significantly lower risk of distant metastasis (OR: 0.348, 95% CI: 0.136–0.891, *p* = 0.024) in the patients harboring wild-type *EGFR* (*n* = 108; Table 3). Patients with the GG genotype were taken as a reference, and the existence of at least one carrier allele in FGFR4 rs351855 had a significantly reduced risk for distant metastasis in all patients (*n* = 277, OR = 0.523, 95% CI = 0.304–0.899, *p* = 0.018; Table 4). In the *EGFR* wild-type group, a significantly reduced presence of distant metastasis was also observed in patients with the *FGFR4* rs351855 A genotype (GA and AA) compared with those with GG homozygotes (*n* = 108, OR = 0.296, 95% CI = 0.116–0.751, *p* = 0.008; Table 4). In addition, no significant association was found between the other two FGFR4 polymorphisms and the clinicopathological characteristics (data not shown).

### 3.4. Clinical Relevance of FGFR4 Levels in Lung Adenocarcinoma Patients with the Wild-Type EGFR Gene Obtained from the Cancer Genome Atlas (TCGA) Databases

**Figure 1:**
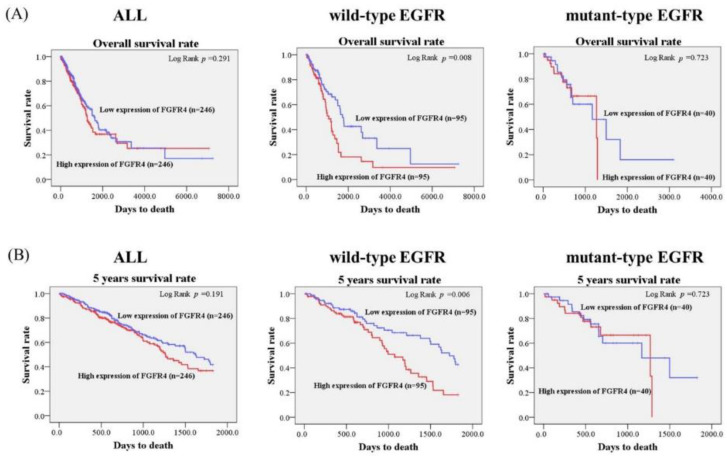
Association of FGFR4 mRNA level and survival rates in lung adenocarcinoma patients from The Cancer Genome Atlas (TCGA) database. (**A**) The effects of FGFR4 mRNA expression on the overall survival of patients with lung adenocarcinoma were evaluated with the Kaplan-Meier plot using the Log-rank test. (**B**) The effects of FGFR4 mRNA expression on the five-year survival of patients with lung adenocarcinoma were evaluated with the Kaplan-Meier plot using the Log-rank test.

Considering the potential effects of *FGFR4* expression levels in the clinicopathological characteristics in lung adenocarcinoma, we further analyzed the correlations between the *FGFR4* expression level and the survival rate in 492 lung adenocarcinoma patients from The Cancer Genome Atlas (TCGA) database. In the TCGA database, 380 patients were early stage (stage I + II). A total of 163 and 24 patients were lymph node metastasis (33.1%) and distant metastasis (4.88%), respectively. As shown in Figure 1, we found that patients in wild-type *EGFR* lung adenocarcinoma with higher *FGFR4* expression had shorter overall survival times and five-years survival times compared with those with lower *FGFR4* expression (*p* = 0.008; Figure 1A; *p* = 0.006; Figure 1B). No significant differences in the *FGFR4* expression and survival rates were observed in all patients with lung adenocarcinoma and in lung adenocarcinoma patients with mutant-type *EGFR*. Therefore, the expression level of *FGFR4* may provide clinical significance for the survival rate of lung adenocarcinoma patients with wild-type *EGFR*.

## 4. Discussion

In the present study, we found no significantly different frequencies of the *FGFR4* SNPs rs2011077, rs351855, rs7708357, and rs1966265 in patients with wild-type EGFR and mutant-type *EGFR* lung adenocarcinoma. The existence of at least one allele of the rs2011077 C genotype or the rs351855 A genotype was associated with a significantly reduced presence of distant metastasis in the *EGFR* wild-type group. The data indicate that both *FGFR4* SNPs rs2011077 and rs351855 may be associated with reduced presence of distant metastasis in Taiwanese patients with lung adenocarcinoma, especially those with the wild-type *EGFR* gene. *FGFR4* SNPs may help in identifying patient subgroups at low-risk for tumor metastasis, among carriers of lung adenocarcinoma bearing wild-type *EGFR*.

Previous studies have shown that the Asia-Pacific patients with lung adenocarcinoma had the higher *EGFR* mutation frequency (47% (5958/12819)) and the Oceania patients with lung adenocarcinoma showed the lower *EGFR* mutation frequency (12% (69/570)) [41]. *EGFR* mutations have been found to occur more frequently in the female and nonsmoking populations and mainly occur in patients with lung adenocarcinoma in the Asian population [4,41,42]. Hsu et al., reported that the *EGFR* mutation rate in Taiwan is higher than 50%, based on data from the National Taiwan Lung Cancer Registry [2]. Consistent with these findings, we noted a higher proportion of women, never-smokers, and cases of well or moderately differentiated tumors in the collected samples. Moreover, most of the specimens were positive for *EGFR* mutations. Two *EGFR* mutations, an in-frame deletion within exon 19 and the L858R point mutation in exon 22, account for approximately 90% of overall *EGFR* mutations in lung adenocarcinoma [43,44]. We examined the exons 18–21 of the *EGFR* gene using direct sequencing to examine *EGFR* mutations in this study. Indeed, a high frequency of *EGFR* mutations (60%) was found in our cohort. No significant association between the four *FGFR4* SNPs and *EGFR* wild-type and *EGFR* mutant-type lung adenocarcinoma was found after adjustment for gender, cigarette smoking, and cell differentiation.

Increasing evidence indicates that upregulation of *FGFR4* expression levels participate in tumorigenesis and cancer progression; thus, *FGFR4* has been proven as a therapeutic target for several cancers [10]. Using in silico analysis of the TCGA database, we found that lung adenocarcinoma patients bearing wild-type *EGFR* with the higher *FGFR4* expression level is correlated with poor overall survival rates and five-years survival rates compared with those with lower *FGFR4* expression patients. The increased *FGFR4* expression is correlated with worse overall survival in head and neck squamous cell carcinoma [13]. Moreover, *FGFR4* rs351855 SNP is associated with the increased *FGFR4* protein expression and a worse prognosis [15]. However, the previous study has found that *FGFR4* mRNA expression levels are not associated with *FGFR4* genotypes in healthy lungs of lung cancer patients [32]. A further study is the association between various *FGFR4* SNPs and *FGFR4* expression levels in lung adenocarcinoma.

We observed that the *FGFR4* SNP rs2011077 T/C was significantly inversely associated with distant metastasis. In addition, after the patients were stratified by the *EGFR* status, both *FGFR4* SNPs rs2011077 T/C and rs351855 G/A were found to be inversely associated with distant metastasis. The *FGFR4* SNP rs351855 G/A, which causes a substitution of glycine for arginine at codon 388 (G388R), has been proven to be related to worse prognosis in many cancers. Fang et al. revealed that the *FGFR4* rs351855 A genotype is correlated with longer overall survival and reduced cancer risk in patients with Stage III (A + B) or IV non-small-cell lung carcinoma [35]. The meta-analytic evidence has demonstrated a modest reduction in the risk of lung cancer [30]. Moreover, no significant association between this *FGFR4* genetic variant and clinical outcomes was found [36]. By contrast, several studies have reported that patients carrying the *FGFR4* rs351855 A genotype had poorer outcomes, including lymph node involvement and reduced overall survival [32,33,34]. Using the molecular approach, the G388R substitution in *FGFR4* was found to cause a conformational change in the *FGFR4* receptor, which then recruits the signal transducer and activator of transcription 3 (STAT3) to the inner cell membrane. The evidence demonstrated that the rs351855 SNP is a significant risk for cancer prognosis and tumor progression [45]. Moreover, a study using *FGFR4* rs351855-A/A-knockin transgenic mice to investigate lung cancer observed a marked decrease in the number of tumor-infiltrating CD8-positive T cells and a significant increase in regulatory T cell proportions compared with those from the G/G-knockin mice [46]. These results suggest that *FGFR4* rs351855 alone could exhibit an immune evasive phenotype of the tumor microenvironment in lung cancer progression [46]. The fact that other studies have reached the opposite conclusion may be due to unknown causes other than *FGFR4* variants. The possibilities of other genetic variations and a greater variety of abnormal signaling molecules are worthy of further investigation.

## 5. Conclusions

Our findings concerning *FGFR4* SNPs rs2011077 and rs351855 may suggest a lower risk of tumor distant metastasis and *FGFR4* gene expression levels may help the prediction of survival rates in Taiwanese lung adenocarcinoma patients, especially those bearing the wild-type *EGFR* gene. The potential mechanisms involved in dysregulated *FGFR4* signaling cascades in Taiwanese patients with lung adenocarcinoma, particularly those with the wild-type *EGFR* gene, warrant further exploration. Further large-scale prospective studies are required to verify the significance of *FGFR4* genetic polymorphisms in lung adenocarcinoma.

## Figures and Tables

**Table 1:** Clinical characteristics in lung adenocarcinoma patients with the epidermal growth factor receptor (*EGFR*) wild-type or mutation-type.

Subject Characteristics	Wild-Type (*n* = 108)	Mutation-Type (*n* = 169)	*p*-Value
**Age, *n* (%)**			
Mean ± SD (years)	65.52 ± 13.55	65.76 ± 13.57	0.817
**Gender, *n* (%)**			
Male	65 (60.2%)	60 (35.5%)	<0.001
Female	43 (39.8%)	109 (64.5%)	
**Cigarette smoking, *n* (%)**			
Non-smoker	48 (44.4%)	131 (77.5%)	<0.001
Ever-smoker	60 (55.6%)	38 (22.5%)	
**Stage, *n* (%)**			
I + II	26 (24.1%)	47 (27.8%)	0.491
III + IV	82 (75.9%)	122 (72.2%)	
**Tumor T status, *n* (%)**			
T1 + T2	60 (55.6%)	108 (63.9%)	0.165
T3 + T4	48 (44.4%)	61 (36.1%)	
**Lymph node status, *n* (%)**			
Negative	28 (25.9%)	54 (32.0%)	0.284
Positive	80 (74.1%)	115 (68.0%)	
**Distant Metastasis, *n* (%)**			
Negative	54 (50.0%)	80 (47.3%)	0.665
Positive	54 (50.0%)	89 (52.7%)	
**Cell differentiation, *n* (%)**			
Well	8 (7.4%)	21 (12.4%)	0.002
Moderately	79 (73.1%)	138 (81.7%)	
Poorly	21 (19.4%)	10 (5.9%)	

Categorical data: *n* (%); continuous variables: Mean + standard deviation (SD); Mann-Whitney U test or Fisher’s exact test was used to evaluate the comparisons between *EGFR* wild-type and mutation-type in lung adenocarcinoma patients. *p*-value < 0.05 was defined as statistical significant.

**Table 2:** Distribution frequency of fibroblast growth factor receptor 4 (*FGFR4*) genotypes of patients with lung adenocarcinoma and multiple logistic regression analysis of *EGFR* mutation association.

Genotype SNP	Wild-Type (*n* = 108)	Mutation-Type (*n* = 169)	AOR (95% CI)	*p*-Value
**rs2011077**				
TT	26 (24.1%)	46 (27.2%)	1.00	
TC	58 (53.7%)	78 (46.2%)	0.86 (0.46–1.60)	0.625
CC	24 (22.2%)	45 (26.6%)	1.16 (0.56–2.42)	0.684
TC + CC	82 (75.9%)	123 (72.8%)	0.95 (0.52–1.71)	0.858
**rs351855**				
GG	28 (25.9%)	48 (28.4%)	1.00	
GA	59 (54.6%)	80 (47.3%)	0.95 (0.51–1.76)	0.870
AA	21 (19.5%)	41 (24.3%)	1.25 (0.59–2.63)	0.561
GA + AA	80 (74.1%)	121 (71.6%)	1.03 (0.58–1.85)	0.914
**rs7708357**				
GG	103 (95.4%)	165 (97.6%)	1.00	
GA	5 (4.6%)	3 (1.8%)	0.52 (0.11–2.43)	0.402
AA	0 (0.0%)	1 (0.6%)		
GA + AA	5 (4.6%)	4 (2.4%)	0.64 (0.15–2.67)	0.535
**rs1966265**				
AA	27 (25.0%)	44 (26.0%)	1.00	
AG	58 (53.7%)	82 (48.5%)	0.97 (0.52–1.82)	0.935
GG	23 (21.3%)	43 (25.5%)	1.31 (0.62–2.74)	0.482
AG + GG	81 (75.0%)	125 (74.0%)	1.07 (0.59–1.93)	0.826

The AORs with 95% CIs were estimated by multiple logistic regression models after controlling for age and gender. Abbreviations: SNP: Single nucleotide polymorphism; AOR: Adjusted odds ratio; CI: Confidence interval.

**Table 3:** Clinicopathologic characteristics of lung adenocarcinoma patients with *EGFR* mutation, stratified by polymorphic genotypes of *FGFR4* rs2011077.

Variable	ALL (*n* = 277)			*EGFR* Wild-Type (*n* = 108)			*EGFR* Mutation (*n* = 169)		
	TT (*n* = 72)	TC + CC (*n* = 205)	*p*-Value	TT (*n* = 26)	TC + CC (*n* = 82)	*p*-Value	TT (*n* = 46)	TC + CC (*n* = 123)	*p-*Value
**Stages**									
I+II	19 (26.4%)	54 (26.3%)	*p* = 0.994	5 (19.2%)	21 (25.6%)	*p* = 0.507	14 (30.4%)	33 (26.8%)	*p* = 0.642
III+IV	53 (73.6%)	151 (73.7%)		21 (80.8%)	61 (74.4%)		32 (69.6%)	90 (73.2%)	
**Tumor T status**									
T1+T2	40 (55.6%)	128 (62.4%)	*p* = 0.304	11 (42.3%)	49 (59.8%)	*p* = 0.119	29 (63.0%)	79 (64.2%)	*p* = 0.887
T3+T4	32 (44.4%)	77 (37.6%)		15 (57.7%)	33 (40.2%)		17 (37.0%)	44 (35.8%)	
**Lymph node status**									
Negative	23 (31.9%)	59 (28.8%)	*p* = 0.613	6 (23.1%)	22 (26.8%)	*p* = 0.704	17 (37.0%)	37 (30.1%)	*p* = 0.394
Positive	49 (68.1%)	146 (71.2%)		20 (76.9%)	58 (73.2%)		29 (63.0%)	86 (69.9%)	
**Distant metastasis**									
Negative	28 (38.9%)	106 (51.7%)	*p* = 0.061	8 (30.8%)	46 (56.1%)	***p* = 0.024 ^a^**	20 (43.5%)	60 (48.8%)	*p* = 0.539
Positive	44 (61.1%)	99 (48.3%)		18 (69.2%)	36 (43.9%)		26 (56.5%)	63 (51.2%)	
**Cell differentiation**									
Well/ Moderately	65 (90.3%)	181 (88.3%)	*p* = 0.646	20 (76.9%)	67 (81.7%)	*p* = 0.591	45 (97.8%)	114 (92.7%)	*p* = 0.207
Poorly	7 (9.7%)	24 (11.7%)		6 (23.1%)	15 (18.3%)		1 (2.2%)	9 (7.3%)	

^a^ OR (95% CI):0.348 (0.136–0.891).

**Table 4:** Clinicopathologic characteristics of lung adenocarcinoma patients with *EGFR* mutation, stratified by polymorphic genotypes of *FGFR4* rs351855.

Variable	ALL (*n* = 277)			*EGFR* Wild-Type (*n* = 108)			*EGFR* Mutation (*n* = 169)		
	GG (*n* = 76)	GA + AA (*n* = 201)	*p*-Value	GG (*n* = 28)	GA + AA (*n* = 80)	*p*-Value	GG (*n* = 48)	GA + AA (*n* =121)	*p*-Value
**Stages**									
I + II	19 (25.0%)	54 (26.9%)	*p* = 0.753	5 (17.9%)	21 (26.2%)	*p* = 0.371	14 (29.2%)	33 (27.3%)	*p* = 0.804
III + IV	57 (75.0%)	151 (73.1%)		23 (82.1%)	59 (73.8%)		34 (70.8%)	88 (72.7%)	
**Tumor T status**									
T1 + T2	41 (53.9%)	127 (63.2%)	*p* = 0.160	12 (42.9%)	48 (60.0%)	*p* = 0.116	29 (60.4%)	79 (65.3%)	*p* = 0.552
T3 + T4	35 (46.1%)	74 (36.8%)		16 (57.1%)	32 (40.0%)		19 (39.6%)	42 (34.7%)	
**Lymph node status**									
Negative	23 (30.3%)	59 (29.4%)	*p* = 0.882	6 (21.4%)	22 (27.5%)	*p* = 0.528	17 (35.4%)	37 (30.6%)	*p* = 0.543
Positive	53 (69.7%)	142 (70.6%)		22 (78.6%)	58 (72.5%)		31 (64.6%)	84 (69.4%)	
**Distant metastasis**									
Negative	28 (38.9%)	106 (52.7%)	***p* = 0.018 ^a^**	8 (28.6%)	46 (57.5%)	***p* = 0.008 ^b^**	20 (41.7%)	60 (49.6%)	*p* = 0.352
Positive	48 (63.2%)	99 (47.3%)		20 (71.4%)	34 (42.5%)		28 (58.3%)	61 (50.4%)	
**Cell differentiation**									
Well/Moderately	69 (90.8%)	181 (88.1%)	*p* = 0.520	22 (78.6%)	65 (81.2%)	*p* = 0.758	47 (97.9%)	112 (92.6%)	*p* = 0.183
Poorly	7 (9.2%)	24 (11.9%)		6 (21.4%)	15 (18.8%)		1 (2.1%)	9 (7.4%)	

^a^ OR (95% CI):0.523 (0.304–0.899); ^b^ OR (95% CI):0.296 (0.116–0.751).

## References

[1] Siegel R.L., Miller K.D., Jemal A. (2019). Cancer statistics, 2019. CA A Cancer J. Clin..

[2] Hsu C.H., Tseng C.H., Chiang C.J., Hsu K.H., Tseng J.S., Chen K.C., Wang C.L., Chen C.Y., Yen S.H., Chiu C.H. (2016). Characteristics of young lung cancer: Analysis of Taiwan's nationwide lung cancer registry focusing on epidermal growth factor receptor mutation and smoking status. Oncotarget.

[3] Yang S.Y., Yang T.Y., Chen K.C., Li Y.J., Hsu K.H., Tsai C.R., Chen C.Y., Hsu C.P., Hsia J.Y., Chuang C.Y. (2011). EGFR l858r mutation and polymorphisms of genes related to estrogen biosynthesis and metabolism in never-smoking female lung adenocarcinoma patients. Clin. Cancer Res..

[4] Shi Y., Au J.S., Thongprasert S., Srinivasan S., Tsai C.M., Khoa M.T., Heeroma K., Itoh Y., Cornelio G., Yang P.C. (2014). A prospective, molecular epidemiology study of EGFR mutations in asian patients with advanced non-small-cell lung cancer of adenocarcinoma histology (pioneer). J. Thorac. Oncol..

[5] Li S., Choi Y.L., Gong Z., Liu X., Lira M., Kan Z., Oh E., Wang J., Ting J.C., Ye X. (2016). Comprehensive characterization of oncogenic drivers in asian lung adenocarcinoma. J. Thorac. Oncol..

[6] Babina I.S., Turner N.C. (2017). Advances and challenges in targeting fgfr signalling in cancer. Nat. Rev. Cancer.

[7] Tiong K.H., Mah L.Y., Leong C.O. (2013). Functional roles of fibroblast growth factor receptors (FGFRS) signaling in human cancers. Apoptosis.

[8] Deng N., Zhou H., Fan H., Yuan Y. (2017). Single nucleotide polymorphisms and cancer susceptibility. Oncotarget.

[9] Quintanal-Villalonga A., Ferrer I., Molina-Pinelo S., Paz-Ares L. (2019). A patent review of FGFR4 selective inhibition in cancer (2007–2018). Expert Opin. Ther. Pat..

[10] Lang L., Teng Y. (2019). Fibroblast growth factor receptor 4 targeting in cancer: New insights into mechanisms and therapeutic strategies. Cells.

[11] Ma Z., Tsuchiya N., Yuasa T., Inoue T., Kumazawa T., Narita S., Horikawa Y., Tsuruta H., Obara T., Saito M. (2008). Polymorphisms of fibroblast growth factor receptor 4 have association with the development of prostate cancer and benign prostatic hyperplasia and the progression of prostate cancer in a Japanese population. Int. J. Cancer.

[12] FitzGerald L.M., Karlins E., Karyadi D.M., Kwon E.M., Koopmeiners J.S., Stanford J.L., Ostrander E.A. (2009). Association of FGFR4 genetic polymorphisms with prostate cancer risk and prognosis. Prostate Cancer Prostatic Dis..

[13] Wimmer E., Ihrler S., Gires O., Streit S., Issing W., Bergmann C. (2019). Fibroblast growth factor receptor 4 single nucleotide polymorphism gly388arg in head and neck carcinomas. World J. Clin. Oncol..

[14] Farnebo L., Tiefenbock K., Ansell A., Thunell L.K., Garvin S., Roberg K. (2013). Strong expression of survivin is associated with positive response to radiotherapy and improved overall survival in head and neck squamous cell carcinoma patients. Int. J. Cancer.

[15] Wei W., You Z., Sun S., Wang Y., Zhang X., Pang D., Jiang Y. (2018). Prognostic implications of fibroblast growth factor receptor 4 polymorphisms in primary breast cancer. Mol. Carcinog..

[16] Chen L., Qi H., Zhang L., Li H., Shao J., Chen H., Zhong M., Shi X., Ye T., Li Q. (2018). Effects of FGFR gene polymorphisms on response and toxicity of cyclophosphamide-epirubicin-docetaxel-based chemotherapy in breast cancer patients. BMC Cancer.

[17] Jiang Y., Sun S., Wei W., Ren Y., Liu J., Pang D. (2015). Association of FGFR3 and FGFR4 gene polymorphisms with breast cancer in chinese women of heilongjiang province. Oncotarget.

[18] Marme F., Werft W., Benner A., Burwinkel B., Sinn P., Sohn C., Lichter P., Hahn M., Schneeweiss A. (2010). FGFR4 arg388 genotype is associated with pathological complete response to neoadjuvant chemotherapy for primary breast cancer. Ann. Oncol..

[19] Thussbas C., Nahrig J., Streit S., Bange J., Kriner M., Kates R., Ulm K., Kiechle M., Hoefler H., Ullrich A. (2006). FGFR4 arg388 allele is associated with resistance to adjuvant therapy in primary breast cancer. J. Clin. Oncol..

[20] Marme F., Hielscher T., Hug S., Bondong S., Zeillinger R., Castillo-Tong D.C., Sehouli J., Braicu I., Vergote I., Isabella C. (2012). Fibroblast growth factor receptor 4 gene (FGFR4) 388arg allele predicts prolonged survival and platinum sensitivity in advanced ovarian cancer. Int. J. Cancer.

[21] Yang Y., Zhou Y., Lu M., An Y., Li R., Chen Y., Lu D.-R., Jin L., Zhou W.-P., Qian J. (2012). Association between fibroblast growth factor receptor 4 polymorphisms and risk of hepatocellular carcinoma. Mol. Carcinog..

[22] Chen T.H., Yang S.F., Liu Y.F., Lin W.L., Han C.P., Wang P.H. (2018). Association of fibroblast growth factor receptor 4 genetic polymorphisms with the development of uterine cervical cancer and patient prognosis. Reprod. Sci..

[23] Tsay M.D., Hsieh M.J., Lee C.Y., Wang S.S., Chen C.S., Hung S.C., Lin C.Y., Yang S.F. (2019). Involvement of FGFR4 gene variants on the clinicopathological severity in urothelial cell carcinoma. Int. J. Environ. Res. Public Health.

[24] Tateno T., Asa S.L., Zheng L., Mayr T., Ullrich A., Ezzat S. (2011). The FGFR4-g388r polymorphism promotes mitochondrial stat3 serine phosphorylation to facilitate pituitary growth hormone cell tumorigenesis. PLoS Genet..

[25] Ezzat S., Wang R., Pintilie M., Asa S.L. (2017). FGFR4 polymorphic alleles modulate mitochondrial respiration: A novel target for somatostatin analog action in pituitary tumors. Oncotarget.

[26] Morimoto Y., Ozaki T., Ouchida M., Umehara N., Ohata N., Yoshida A., Shimizu K., Inoue H. (2003). Single nucleotide polymorphism in fibroblast growth factor receptor 4 at codon 388 is associated with prognosis in high-grade soft tissue sarcoma. Cancer.

[27] Dutra R.L., de Carvalho M.B., Dos Santos M., Mercante A.M., Gazito D., de Cicco R., Group G., Tajara E.H., Louro I.D., da Silva A.M. (2012). FGFR4 profile as a prognostic marker in squamous cell carcinoma of the mouth and oropharynx. PLoS ONE.

[28] Choi K.Y., Rho Y.S., Kwon K.H., Chung E.J., Kim J.H., Park I.S., Lee D.J. (2012). ECRG1 and FGFR4 single nucleotide polymorphism as predictive factors for nodal metastasis in oral squamous cell carcinoma. Cancer Biomark.

[29] Chou C.H., Hsieh M.J., Chuang C.Y., Lin J.T., Yeh C.M., Tseng P.Y., Yang S.F., Chen M.K., Lin C.W. (2017). Functional FGFR4 gly388arg polymorphism contributes to oral squamous cell carcinoma susceptibility. Oncotarget.

[30] Xiong S.W., Ma J., Feng F., Fu W., Shu S.R., Ma T., Wu C., Liu G.C., Zhu J. (2017). Functional FGFR4 gly388arg polymorphism contributes to cancer susceptibility: Evidence from meta-analysis. Oncotarget.

[31] Xu W., Li Y., Wang X., Chen B., Wang Y., Liu S., Xu J., Zhao W., Wu J. (2010). FGFR4 transmembrane domain polymorphism and cancer risk: A meta-analysis including 8555 subjects. Eur. J. Cancer.

[32] Spinola M., Leoni V., Pignatiello C., Conti B., Ravagnani F., Pastorino U., Dragani T.A. (2005). Functional FGFR4 gly388arg polymorphism predicts prognosis in lung adenocarcinoma patients. J. Clin. Oncol..

[33] Falvella F.S., Frullanti E., Galvan A., Spinola M., Noci S., De Cecco L., Nosotti M., Santambrogio L., Incarbone M., Alloisio M. (2009). FGFR4 gly388arg polymorphism may affect the clinical stage of patients with lung cancer by modulating the transcriptional profile of normal lung. Int. J. Cancer.

[34] Sasaki H., Okuda K., Kawano O., Yukiue H., Yano M., Fujii Y. (2008). Fibroblast growth factor receptor 4 mutation and polymorphism in japanese lung cancer. Oncol. Rep..

[35] Fang H.M., Tian G., Zhou L.J., Zhou H.Y., Fang Y.Z. (2013). FGFR4 genetic polymorphisms determine the chemotherapy response of chinese patients with non-small cell lung cancer. Acta Pharmacol. Sin..

[36] Matakidou A., El Galta R., Rudd M.F., Webb E.L., Bridle H., Eisen T., Houlston R.S. (2007). Further observations on the relationship between the FGFR4 gly388arg polymorphism and lung cancer prognosis. Br. J. Cancer.

[37] Goldstraw P., Chansky K., Crowley J., Rami-Porta R., Asamura H., Eberhardt W.E., Nicholson A.G., Groome P., Mitchell A., Bolejack V. (2016). The iaslc lung cancer staging project: Proposals for revision of the tnm stage groupings in the forthcoming (eighth) edition of the tnm classification for lung cancer. J. Thorac. Oncol..

[38] Chou Y.-E., Hsieh M.-J., Hsin C.-H., Chiang W.-L., Lai Y.-C., Lee Y.-H., Huang S.-C., Yang S.-F., Lin C.-W. (2014). CD44 gene polymorphisms and environmental factors on oral cancer susceptibility in Taiwan. PLoS ONE.

[39] Sheu M.J., Hsieh M.J., Chiang W.L., Yang S.F., Lee H.L., Lee L.M., Yeh C.B. (2015). Fibroblast growth factor receptor 4 polymorphism is associated with liver cirrhosis in hepatocarcinoma. PLoS ONE.

[40] Hua K.T., Liu Y.F., Hsu C.L., Cheng T.Y., Yang C.Y., Chang J.S., Lee W.J., Hsiao M., Juan H.F., Chien M.H. (2017). 3'utr polymorphisms of carbonic anhydrase ix determine the mir-34a targeting efficiency and prognosis of hepatocellular carcinoma. Sci. Rep..

[41] Midha A., Dearden S., McCormack R. (2015). Egfr mutation incidence in non-small-cell lung cancer of adenocarcinoma histology: A systematic review and global map by ethnicity (mutmapii). Am. J. Cancer Res..

[42] de Groot P.M., Wu C.C., Carter B.W., Munden R.F. (2018). The epidemiology of lung cancer. Transl. Lung Cancer Res..

[43] Rosell R., Moran T., Queralt C., Porta R., Cardenal F., Camps C., Majem M., Lopez-Vivanco G., Isla D., Provencio M. (2009). Screening for epidermal growth factor receptor mutations in lung cancer. N. Engl. J. Med..

[44] Hong W., Wu Q., Zhang J., Zhou Y. (2019). Prognostic value of EGFR 19-del and 21-l858r mutations in patients with non-small cell lung cancer. Oncol. Lett..

[45] Ulaganathan V.K., Sperl B., Rapp U.R., Ullrich A. (2015). Germline variant FGFR4  p.G388r exposes a membrane-proximal stat3 binding site. Nature.

[46] Kogan D., Grabner A., Yanucil C., Faul C., Ulaganathan V.K. (2018). Stat3-enhancing germline mutations contribute to tumor-extrinsic immune evasion. J. Clin. Investig..

